# Insights into Interfacial and Bulk Transport Phenomena Affecting Proton Exchange Membrane Water Electrolyzer Performance at Ultra‐Low Iridium Loadings

**DOI:** 10.1002/advs.202102950

**Published:** 2021-09-26

**Authors:** Xiong Peng, Pongsarun Satjaritanun, Zachary Taie, Luke Wiles, Alex Keane, Christopher Capuano, Iryna V. Zenyuk, Nemanja Danilovic

**Affiliations:** ^1^ Energy Storage and Distributed Resources Division Lawrence Berkeley National Laboratory Berkeley CA 94720 USA; ^2^ Department of Material Science and Engineering University of California Irvine Irvine CA 92697 USA; ^3^ Oregon State University School of Mechanical, Industrial, and Manufacturing Engineering Bend OR 97702 USA; ^4^ Nel Hydrogen/Proton Onsite Wallingford CT 06492 USA

**Keywords:** electrolysis, hydrogen, iridium, PEMWE, porous transport layer, ultra‐low loading, X‐ray computed tomography

## Abstract

Interfacial and bulk properties between the catalyst layer and the porous transport layer (PTL) restrict the iridium loading reduction for proton exchange membrane water electrolyzers (PEMWEs), by limiting their mass and charge transport. Using titanium fiber PTLs of varying thickness and porosity, the bulk and interface transport properties are investigated, correlating them to PEMWEs cell performance at ultra‐low Ir loadings of ≈0.05 mg_Ir_ cm^−2^. Electrochemical experiments, tomography, and modeling are combined to study the bulk and interfacial impacts of PTLs on PEMWE performance. It is found that the PEMWE performance is largely dependent on the PTL properties at ultra‐low Ir loadings; bulk structural properties are critical to determine the mass transport and Ohmic resistance of PEMWEs while the surface properties of PTLs are critical to govern the catalyst layer utilization and electrode kinetics. The PTL‐induced variation in kinetic and mass transport overpotential are on the order of ≈40 and 60 mV (at 80 A mg_Ir_^−1^), respectively, while a nonnegligible 35 mV (at 3 A cm^−2^) difference in Ohmic overpotential. Thus at least 150 mV improvement in PEMWE performance can be achieved through PTL structural optimization without membrane thickness reduction or advent of new electrocatalysts.

## Graphical abstract

The effect of interfacial and bulk properties of porous transport layers (PTL) on proton exchange membrane water electrolyzers (PEMWEs) performance, mass, and charge transport limitations at ultra‐low iridium catalyst loadings is explored. The result provides insights into optimal PTL design: high porosity and low tortuosity in the bulk and intermediate porosity at the interface for PEMWEs operations.

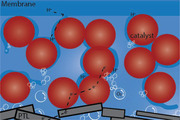

## 1 Introduction

Proton exchange membrane water electrolyzers (PEMWEs) are increasingly being considered as an essential technology to integrate the growing share of renewable power into many energy sectors, since they convert renewable electricity into hydrogen, which is a stable, clean energy carrier, and commodity chemical that can effectively displace fossil fuels in food production and manufacturing.^[^ ^1^, ^2^ ^]^ PEMWEs offer many advantages over other electrolysis chemistries, namely, KOH and anion exchange membrane, due to PEMWE's superiority in coupling with renewable, intermittent sources of cheap electrons:^[^ ^3^, ^4^ ^]^ high current density operation resulting in small footprints, high turn‐down ratios, pressurized hydrogen delivery without the need for dealing with high pressure oxygen, and quick start‐up and response times.^[^ ^3^, ^5^ ^]^ The constraint in widespread adoption of PEMWEs has been driven by both the electrolyzer capital cost and electricity feedstock cost, which needs to be reduced substantially to make PEMWEs a techno‐economically favorable contender to meet the growing demand of renewable electricity deployment.^[^ ^6^, ^7^, ^8^ ^]^

**Figure 1:**
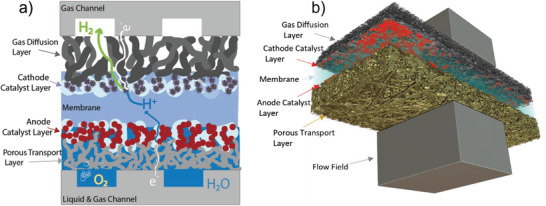
a) Detail of water and oxygen flow within the anode catalyst and porous transport layers. b) Volume‐rendered XCT image of the membrane electrode assembly with flow channel/current collector.

Historically, the high cost of hydrogen from electrolysis was predominantly dictated by the high cost of front of the meter grid electricity. In the new energy landscape, capturing significantly cheaper, intermittent renewable electrons can reduce to the cost of hydrogen to make it competitive with steam methane reforming.^[^ ^6^ ^]^ However, a reduction of capital cost for PEMWEs will determine the feasibility of installing and deploying PEMWEs at the terawatt (TW) scale necessary.^[^ ^3^, ^9^ ^]^ The PEMWE stack capital cost is dominated by the cost of the membrane electrode assembly (MEA, **Figure** **1**), which consists of a perfluorsulfonic membrane, anode and cathode catalyst, and diffusion layers.^[^ ^10^ ^]^ The anode side of the cell where the titanium porous transport layer (PTL) and iridium‐based catalysts dominate the technoeconomics with a combination of overengineering of Ir and Ti in terms of quantity, and underengineering resulting in poor utilization and fluid dynamics.^[^ ^11^, ^12^ ^]^ Iridium‐based catalysts are the only viable catalysts for the oxygen evolution reaction,^[^ ^13^, ^14^ ^]^ the scarcity of which contributes to the high capital cost. Ir is the least abundant element on the earth, requiring a 50‐fold reduction of Ir‐specific power density (g_Ir_ kW^−1^), down to 0.01, compared to today's commercial electrolyzers (≈mg_Ir_'s cm^−2^).^[^ ^15^ ^]^ In order to enable the necessary Ir loading reduction down to 0.05–0.1 mg cm^−2^, efficient catalyst utilization is extremely important in PEMWE as less catalytic sites are available.^[^ ^5^ ^]^ The current understanding in literature is that catalyst layer utilization is controlled by a combination of poor catalyst layer electronic conductivity and the poor interfacial area due to porous PTLs requiring microporous layers (MPL).^[^ ^16^, ^17^, ^18^ ^]^ This narrative is at odds with recent work showing that i) oxygen transport through the titanium PTL may be a crucial limiting factor contributing to performance decline,^[^ ^19^, ^20^ ^]^ ii) high performance at ultra‐low loadings without MPLs is possible,^[^ ^5^ ^]^ iii) catalyst layer ionic resistance dominates over electronic resistance when ionomer volume fraction is higher than 20%.^[^ ^21^ ^]^

We hypothesize that beyond improving catalyst layer utilization, the interface and the bulk of the PTL also mediate the two‐phase flow of removing oxygen bubbles from catalyst layer active sites and supplying water for the reaction and to hydrate the membrane and ionomer. In other words, we suspect that the structure–property relationship of the PTL, which mediates the two‐phase flow in the anode side of the cell, is poorly understood and can significantly impact the technoeconomics of PEMWE.^[^ ^22^, ^23^ ^]^ To investigate this, we use the ultra‐low catalyst loading regime (0.05 mg_Ir_ cm^−2^) to sensitize the PEMWE to changes in the PTL itself, to simulate end of life loadings, and to highlight underlying limitations that may lead to unseen degradation pathways such as water starvation and O_2_ removal. We highlight that based on our recent work, this loading still allows better than state‐of‐the‐art performance, meaning it is highly relevant regime.^[^ ^5^ ^]^ Additionally, we recently showed a modified iridium‐based catalyst achieving stable performance after 30 000 cycles.^[^ ^23^ ^]^ Using the ultra‐low loaded MEAs, we then vary the thickness and porosity of sintered titanium fiber‐based PTLs (from Bekaert) and evaluate the resulting PEMWE performance and catalyst utilization. Using a combination of X‐ray computed tomography (XCT) and in‐cell testing to quantify how surface and bulk properties of different PTLs could affect PEMWEs performance, specifically the kinetic, Ohmic, and mass transport overpotentials at ultra‐low Ir loadings. We then quantify the oxygen dynamics within the PTL and its interfaces with the catalyst layer and flow field using a multiphysics model.

## 2 Results

**Figure 2:**
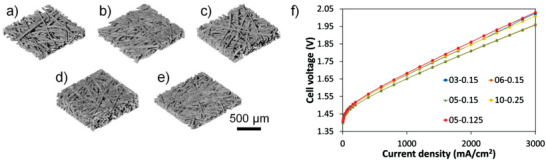
Volume rendering of XCT images of five PTLs: a) 03‐0.15, b) 05‐0.15, c) 06‐0.15, d) 10‐0.25, and e) 05‐0.125. f) Polarization curves of five different PTLs at ultra‐low Ir loading in PEMWEs. 03‐0.15: 0.030 mg_Ir_ cm^−2^, 05‐0.15: 0.035 mg_Ir_ cm^−2^, PTL 06‐0.15: 0.039 mg_Ir_ cm^−2^; 10‐0.25: 0.036 mg_Ir_ cm^−2^, and 05‐0.125: 0.040 mg_Ir_ cm^−2^. Nafion 117 was used as the membrane. Tests were conducted at 80 °C and repeated at least three times for each PTL. Data shown without iR‐correction.

Five different PTLs from Bekaert are selected with Ti fiber diameter of 20 µm. A spanwise strength test was conducted to assess the ability of the PTL to withstand the forces applied under differential pressure conditions; all the PTLs showed adequate mechanical strength (Figure S1, Supporting Information) for electrolysis applications. The surface and bulk morphology and properties of the PTLs were revealed by scanning electron microscopy (SEM; Figure S2, Supporting Information) and more importantly X‐ray CT (**Figure** **2**a–e) and properties are summarized in **Table** **1**. The range of in‐plane and through‐plane porosity, tortuosity, as well as interfacial area allows us to interrogate the crucial impacts of liquid and gas transport during PEMWEs operations (Figures S3–S6, Supporting Information). Porosity and tortuosity relate effective transport properties, *K*_eff_, with bulk transport properties, *K* through the following relation

(1) $$K_{\mathrm{eff}}=K\times \frac{\varepsilon }{\tau }$$

where *ε* is the porosity and *τ* is the tortuosity. The effective transport property can be diffusivity or permeability for transport in pores. The higher the porosity and the lower the tortuosity the better the effective transport properties. Note that effective transport properties become bulk, when porosity and tortuosity is 1. As will be discussed later, the ratio of porosity to tortuosity in this equation is the formation factor.

**Table 1:** Bulk properties of the different PTLs determined by X‐ray CT and image analysis

PTL type	Porosity [%]	Mean pore size/radius [µm]	Through‐plane tortuosity	In‐plane tortuosity	Thickness [µm]
03‐0.15	74.9	28.0	1.27	1.24	155
05‐0.15	60.0	15.4	1.59	1.67	142
06‐0.15	62.1	20.5	1.59	1.78	150
10‐0.25	54.4	13.4	1.75	2.15	246
05‐0.125	55.0	13.5	2.13	2.25	122

The PTLs were integrated in ultra‐low Ir‐loaded PEMWEs for electrochemical measurements. The PEMWEs testing followed a protocol in previous work and was conducted independently for each PTL.^[^ ^5^ ^]^ It is important to point out that the catalyst layers used were fabricated under the same fabrication conditions with minor variations in loadings, which can be considered effectively the same, thus differences in cell performance we observe are largely attributed to different PTL properties. Geometric polarization performance of the ultra‐low Ir‐loaded PEMWEs with five PTLs is summarized in Figure 2f. First, we note that the performance of all five PEMWE, irrespective of PTL and low loading are the highest reported and consistent with our previous work,^[^ ^5^ ^]^ however we see intriguing, substantial differences in performance between different PTLs, as much as 80 mV at 3 A cm^−2^, indicating that the PTL structural differences have an even bigger impact on the cell Ohmic resistance and mass transport resistance than kinetics, as expected.^[^ ^20^ ^]^

## 3 Discussion

**Figure 3:**
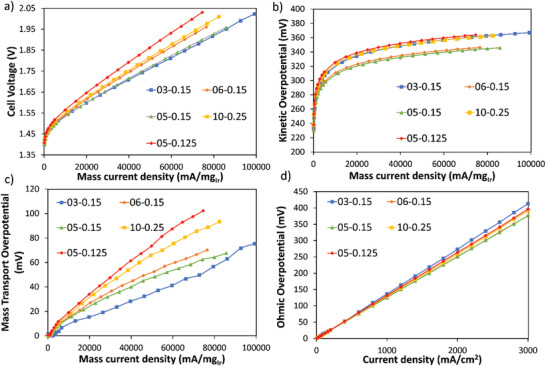
a) PEMWE polarization curves using mass normalized current using different PTLs, b) Kinetic overpotential, c) mass transport overpotential against mass normalized current in PEMWEs using different PTLs, and d) Ohmic overpotential distribution of different PTLs.

**Figure 4:**
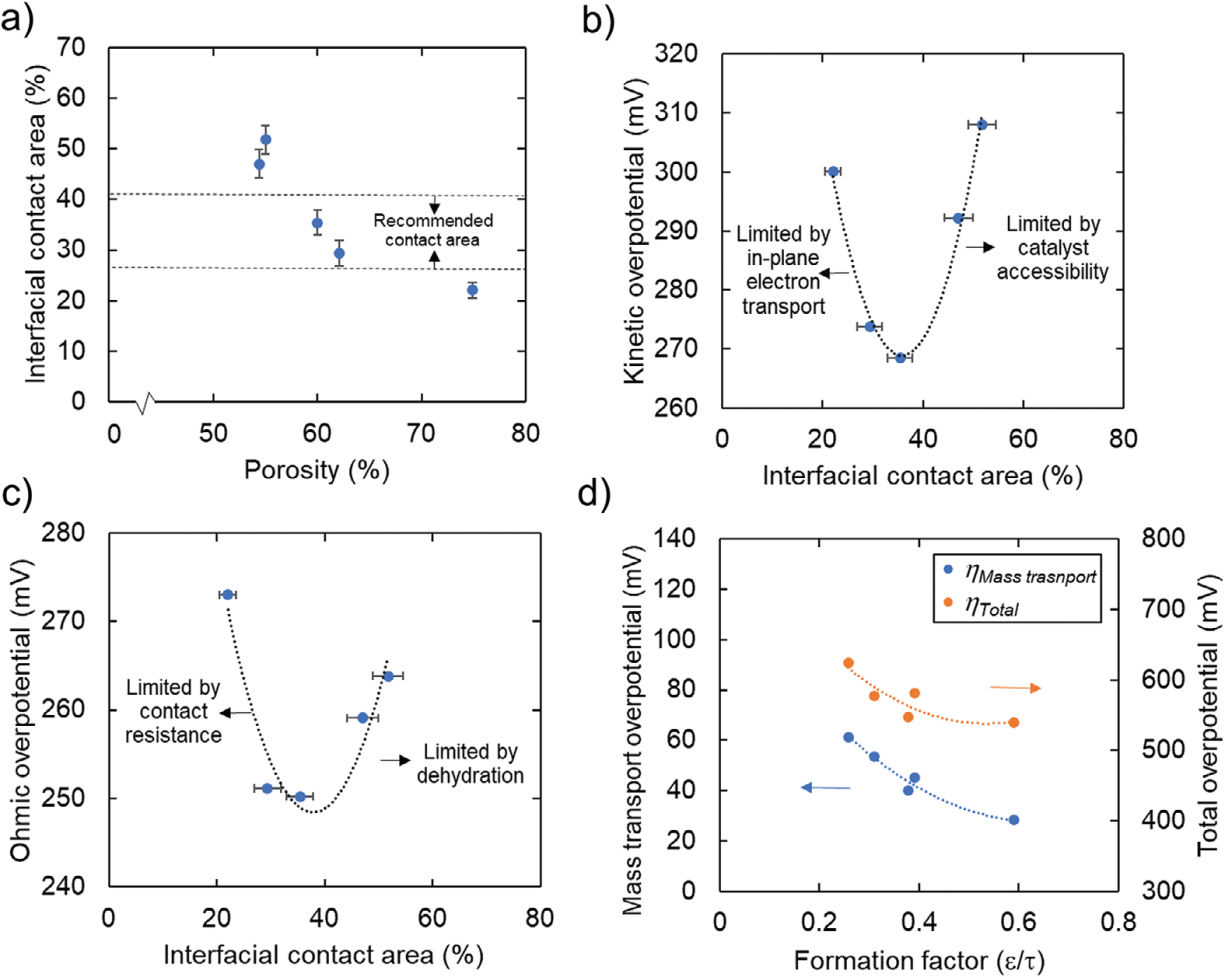
a) Relationship between interfacial contact area and porosity, b) effect of interfacial contact area on the kinetic overpotential, the kinetic overpotentials were selected at 25 A mg_Ir_^−1^, c) Ohmic overpotential as a function of interfacial contact area, where the Ohmic overpotentials were selected at 2 A cm^−2^, and d) effect of formation factor on mass transport overpotential and total overpotential, the mass transport overpotential and total overpotential were selected at 40 A mg_Ir_^−1^.

On a mass normalized basis, the performance variances are even larger for different PTLs, while accounting for variations in ultra‐low catalyst loading (**Figure** **3**a). We extracted the kinetic and mass‐transport overpotential against mass‐based current for the five PTLs (voltage breakdown process in the Supporting Information). Starting with the kinetic overpotentials, shown in Figure 3b, the PTL‐induced variation in kinetic overpotential is on the order of about 40 mV (from 20 to 80 A mg_Ir_^−1^). The dependence of interfacial contact area (Figure S7, Supporting Information) on porosity is shown by **Figure** **4**a, where a linear relation is observed, with increasing PTL porosity there is a decrease in contact area between the PTL and catalyst layer. With the PTL 03‐0.15 at high porosity, there is likely too little interfacial area (only 22%) and the electrode kinetics suffers, since at ultra‐low Ir loading (0.05–0.1 mg cm^−2^), it is extremely challenging to maintain catalyst layer integrity especially during electrolyzer operation,^[^ ^24^ ^]^ therefore, some isolated catalyst islands could form, which significantly reduces catalyst utilization and hurts electrode kinetics in the noncontact area. Under such circumstances, the in‐plane catalyst layer electric conductivity plays a vital role in determining the catalyst utilization. On the other hand, too high of an interfacial area (PTL 10‐0.25 and 05‐0.125 having 47% and 52% contact area, respectively) results in poor site access to the Ir, which is presumably buried directly under too high of a PTL fiber area. Although it has good electric contact with the PTL, it can result in a lack of water access, which leads to unutilized catalyst sites. The optimal kinetics are seen in the two PTLs with intermediate porosities and interfacial areas, 05‐0.15 and 06‐0.15. At intermediate interfacial areas of 35.4% and 29.4%, there is a balance between noncontact areas, reducing the resistance for electrons to freely travel between the PTL and catalyst interfaces, thus resulting in higher catalyst utilization and better electrode kinetics. Figure 4b shows quantitatively the dependence of kinetic overpotential on interfacial contact area, as just discussed the optimal contact area between the PTL and catalyst layer is somewhere around 29–40%. This conclusion is directly at odds with the prescription for the need for microporous layers for PTLs.^[^ ^18^, ^25^ ^]^ Therefore, we emphasize that this conclusion applies to PTLs that have uniform and not graded porosity, as Schuler and co‐workers^[^ ^18^ ^]^ showed the higher the contact area between PTL and catalyst layer, the higher the catalyst utilization, however in that case a microporous layer was used to increase the interfacial density. Here we find, for very dense interfaces catalyst accessibility suffers. The differing conclusions are likely because of the catalyst layer quality and loading. We note that the PEMWE performance in this paper is better by 50 mV at 3 A cm^−2^, while simultaneously decreasing the catalyst loading by two orders of magnitude and increasing the membrane thickness by 2 mil (Figure S8, Supporting Information).^[^ ^18^ ^]^ Compared to other literatures,^[^ ^18^, ^25^, ^26^, ^27^, ^28^, ^29^, ^30^, ^31^, ^32^, ^33^ ^]^ the PEMWEs performance in this work also showed superiority with much lower Ir loadings with thicker membrane (Table S1, Supporting Information).

The bulk transport properties of the PTL should significantly impact the water and O_2_ transport in the PEMWEs, thus governing the mass transport overpotential of the cell.^[^ ^34^ ^]^ On the one side, the oxygen bubbles can change the liquid water permeability within the PTL architecture, on the other side, oxygen bubbles, if not removed efficiently, can create dry areas where no liquid water can assess the catalyst layer, leading to significant mass transport resistance and potentially Ohmic losses from membrane dehydration. The mass transfer overpotential against mass normalized currents is shown in Figure 3c for five PTLs at 40 A mg_Ir_^−1^. The lowest mass transport resistance is seen for the PTL (03‐0.15) with the highest porosity and the least tortuosity, while the worst was in the PTL (05‐0.125) with the highest tortuosity and among the lowest porosity. Although PTL 05‐0.125 is thinner than PTL 10‐0.25 with similar porosity, due to its higher through‐plane tortuosity, it has higher mass transport resistance. This is intuitively understood, the mass transfer overpotential is generally governed by the PTL porosity and through‐plane tortuosity. As the increase of porosity and decrease of through‐plane tortuosity, the resistance for water diffusion toward the catalyst and oxygen bubbles diffusion away from the catalyst layer decreases, thus resulting in a smaller fluid transport overpotential. This finding is a discrepancy compared to previous work by Lopata et al.^[^ ^35^ ^]^ claiming no impact of PTL bulk properties on PEMWE performance. To further interrogate these effects, we introduce the formation factor, defined as the ratio of porosity to tortuosity and when multiplied by *bulk* transport property results in the *effective* transport property, as shown in Equation (1). We observe that the mass transport overpotential, at a fixed utilization of 40 A mg_Ir_^−1^, depends inversely on the formation factor, as shown by Figure 4d. This trend has also been observed at higher current densities (fixed utilization of 60 and 80 A mg_Ir_^−1^), where mass transport resistance is higher, as shown by Figure S9c,d in the Supporting Information. Therefore, the mass transport overpotential is solely dependent on morphological properties of the PTL.

The Ohmic overpotential of PEMWEs is representative of the full cell DC resistance, mainly governed by the contact resistance and ionic transfer resistance within the proton exchange membrane. Since this is directly linked to the geometric area of the cell, not the catalyst mass loading, we therefore choose geometric current density instead of mass normalized current density for comparison. As shown in Figure 3d, there is a nonnegligible 35 mV (at 3 A cm^−2^) difference in Ohmic overpotential difference among the best the 60% porosity PTLs (05‐0.15 and 06‐0.15), and the worst the 78% (03‐0.15). However, it is contact area (local transport) and not the bulk porosity (bulk transport) that seems to dominate, Figure 4c shows the dependence of Ohmic overpotential on PTL‐catalyst layer contact area at 2 A cm^−2^ (see Figure S9a,b in the Supporting Information for 1 A cm^−2^ and 3 A cm^−2^, respectively), similarly to what was observed for kinetic overpotential. We understand the reasoning to be a complex interaction between the O_2_ flux from the active sites competing with allowing water access to the membrane for hydration. For lower contact areas, the contact between the PTL and catalyst layer introduces higher overpotential, whereas for higher contact areas, oxygen removal obstructs water delivery resulting in local membrane dehydration.

**Figure 5:**
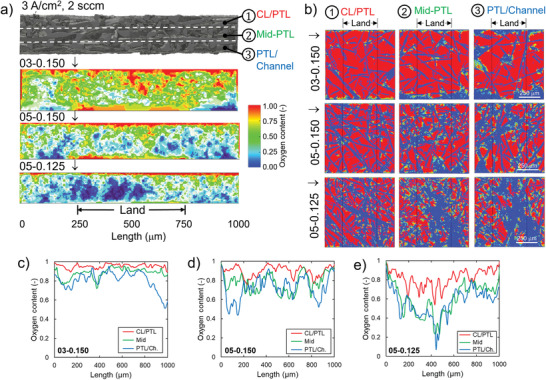
Summary plots detailing the oxygen distribution at interface of CL/PTL, middle interface of PTL, and interface of PTL/flow field channel for the samples of 03‐0.150, 05‐0.150, and 05‐0.125, respectively. a) In‐plane oxygen distribution of these samples, b) through‐plane oxygen distribution at each interface. LBM simulation was conducted at 3 A cm^−2^, and oxygen content profiles comparison within different PTL portions as a function of distance for sample c) 03‐0.150, d) 05‐0.150, and e) 05‐0.125.

We further explore the complex transport in these PTLs using a lattice Boltzmann method (LBM) in conjunction with the PTL tomography, of the oxygen distribution within PTLs at three different regions of catalyst layer/PTL (CL/PTL) interface, middle region of PTL (mid‐PTL), and PTL/flow field channel (PTL/channel) interface at a fixed current density of 3 A cm^−2^ (total oxygen generation rate is the same for all PTLs, **Figure** **5**). The model boundary conditions and O_2_ flux are shown in Figures S10 and S11 in the Supporting Information, respectively. We compare the best‐performing PTLs, 03‐150 (mass normalized) and 05‐150 (geometric area based) and the worst 05‐125 (both mass and geometric). The in‐plane view of oxygen distribution results shows that oxygen is preferentially present in the CL/PTL interface, where it is generated. Oxygen content decreases when transporting through the mid PTL and PTL/channel, respectively, for all five studied PTLs. The result also indicates that PTL structural differences could lead to a dramatic variance in oxygen distribution within the CL/PTL, mid‐PTL, and PTL/channel. The broader and more even oxygen distribution indicates more oxygen transport pathways and less oxygen removal resistance, likely eases water permeation to active sites, which should result in a lower overall mass transport overpotential during PEMWEs operation. Comparing 03‐0.15 (Figure 4a,b top,c) with the lowest and 05‐0.125 (Figure 5a,b bottom,e) with the highest mass transport resistance, respectively, the impact of the PTL structure on the oxygen distribution is clearly evident. A highly porous and low tortuosity PTL achieves the highest oxygen transport pathway across each interface of CL/PTL, mid‐PTL, and PTL/channel as well as through plane. A high tortuosity and low porosity PTL channels O_2_ through narrow bands of preferential pathways, limiting the diffusion of oxygen across the interfaces and through the plane of the PTL. Which strikingly results in no oxygen at the channel/PTL interface, under the land of the channel (Figure 5a bottom). A broader oxygen distribution at the CL/PTL interface also means a higher catalyst utilization, and lower kinetic overpotentials. However, oxygen concentration near the CL is not the only determining factor for the kinetic losses, as already discussed, kinetic overpotential also depends on the CL/PTL contact area and CL in‐plane electric conductivity. Comparing 03‐0.15 (Figure 4a,b top,c) with the highest and 05‐0.15 (Figure 5a,b middle,d) with the lowest kinetic resistance, respectively, the kinetic differences are picked up resulting from the different interfacial contact areas between PTLs (Figure S7, Supporting Information). The trends observed here for 3 A cm^−2^ LBM simulation in terms of uniformity of oxygen distribution, hold for 1 and 2 A cm^−2^ simulations shown in Figures S12 and S13 in the Supporting Information, respectively. At lower current density of 0.5 A cm^−2^ (Figure S14, Supporting Information), all three PTLs show oxygen only in the first half of the PTL (closest to the catalyst layer), however, even at this low current density PTL 05‐0.125 showed significant oxygen depletion in the PTL under the land location, as shown by Figure 5c–e. Figure S15 in the Supporting Information shows the simulations for the other two PTLs at 1 A cm^−2^. Note that the modeling framework assumes that oxygen flux is uniform over the catalyst area in contact with pore space and thus oxygen content at CL/PTL interface is close to 1 (for 03‐0.150 and 05‐0.15), which might be an exaggeration as liquid content of water in this case is 0.05–0.1.

## 4 Conclusion

In summary, our findings indicate that the electrode kinetics of ultra‐low Ir‐loaded PEMWEs are mainly governed by the PTL surface porosity and bulk tortuosity and porosity. Significantly, the PTL surface porosity needs to consider the balance between PTL/catalyst layer contact area and water accessibility for the catalyst layer that is buried under PTL fibers. The PTL bulk properties can significantly impact PEMWEs mass transport resistance by impacting water and oxygen distribution and transport across PTLs. PTLs with high porosity and low tortuosity can promote removal of oxygen and enhance water permeability, therefore offering low mass transport resistance for PEMWEs operation. A PTL with this combination of properties would allow for optimal catalyst layer utilization and two‐phase flow ensuring optimal performance and likely resulting in improved durability as operating in the mass transport‐limited regime is detrimental to long‐term stability.

## 5 Experimental Section

### Catalyst Coated Membrane (CCM) Fabrication

Anode ink was prepared by mixing commercially available iridium oxide (SA = 100, Tanaka) with water, ethanol, and n‐propanol at a ratio of 1:1:2 by volume, and Nafion ionomer solution (5 wt%, Ion Power D521). Ionomer to iridium ratio was maintained to be 0.116 in this study. The anode ink was immersed in an ice bath, sealed with parafilm, and sonicated using a horn sonicator (CEX500, Cole‐Parmer) at 30% of power for 30 min prior to deposition. The cathode ink was prepared by mixing platinum supported by carbon catalyst (45.6 wt% Pt, Tanaka) with equal parts water and n‐propanol, and Nafion ionomer solution (5 wt%, Ion Power D521). The cathode inks were then sonicated at a constant temperature of 10 °C in a bath sonicator for 30 min prior to deposition. The volume of both inks was kept constant during the entire study to minimize the impact of ink preparation on cell performance. The inks were prepared immediately prior to being spray‐deposited onto Nafion N117 membranes using a Sono‐Tek ultrasonic spray coater. The Nafion perfluorosulfonic acid membranes (N117, Ion Power) were prepared by soaking in deionized (DI) water at 90 °C for 1 h and followed by immersion in 0.5 m HNO_3_ (ACS Reagent, Sigma‐Aldrich) for 1 h at room temperature to remove impurities and protonate the sulfonic‐acid groups. Finally, the treated membranes were rinsed three times using DI water to remove excess acid and stored in DI water until catalyst coating is performed. The Sono‐Tek sonication was set to 120 kHz, and the spray deck was modified with a porous aluminum plate and vacuum pump that pulled the membrane flat. The spray deck was held constant at 90 °C. The cathode and anode deposition processes were similar, with spray passes held constant where possible. After spray coating, the precious metal loading was measured using X‐ray fluorescence (XRF) (Bruker M4 Tornado). The XRF was calibrated using commercial standards (Micromatter Technologies Inc.) and calibration curves are shown in the Supporting Information.

### Cell Assembly

CCMs with 5 cm^2^ active area were assembled in single cell hardware (Fuel Cell Technology, FCT) with a graphite single channel serpentine flow field on cathode and platinized titanium parallel flow field on anode. The CCM was rehydrated at room temperature in DI water before it was assembled into the cell. Teflon gaskets were used on anode and cathode, respectively. Carbon paper without microporous layer (Toray 120) was used as cathode gas diffusion layers (GDLs) while the five chosen porous‐transport layers (PTLs) were used on the anode. A platinum coating was used to protect PTL from corrosion by electroplating (applied by NEL Hydrogen). The appropriate thickness polytetrafluoroethylene (McMaster‐Carr) gaskets were used to obtain 20% compression in GDLs, while thickness‐matched gasket was used for the titanium (Ti)‐PTL, verified with pressure films. A torque of 4.5 Nm was applied to the cell to ensure proper sealing.

### Cell Testing

A multichannel potentiostat (VSP300, Biologic) equipped with electrochemical impedance spectroscopy (EIS) and a 20 A booster was used for all electrochemical tests. The test station used was a modified FCT test stand; the modification was an addition of a water recirculation system for the electrolyzer mode testing. Before any electrochemical testing, hot DI water was supplied at 80 °C on anode side to preheat the cell for 30 min, after which, an auxiliary cell heater was used to further heat up the cell to 80 °C. Temperature uniformity across the cell was verified with an IR camera. 100 mL min^−1^ of H_2_ was supplied to the cathode at ambient pressure to ensure a pseudo‐steady reference electrode for electrolyzer operation. Conditioned cyclic voltammetry (CV) was taken via scanning between 1.2 and 2 V at 50 mV s^−1^ for ten cycles before recording polarization curves and electrochemical impedance. The polarization curve was taken by holding at various constant cell currents for 130 s while recording cell voltage. The impedance was measured in a galvanostatic mode by applying an AC current perturbation between 200 kHz and 100 mHz to the cell and measuring its voltage response at each polarization step. The amplitude of the AC current was chosen for each step to obtain a sufficient signal to noise ratio, while keeping the perturbation small enough to ensure a linear system response. The Ir cyclic voltammetry was measured by cycling electrode from 0.05 to 1.2 V at 50 mV s^−1^ at 80 °C with DI water and H_2_ fed to anode and cathode, respectively.

### Overpotential Analysis^[1]^

The cell voltage *E*_cell_ is composed of the sum of the reversible cell potential $E_{\mathrm{rev}}^{0}$ and the three main overpotential *η*_i_

(2) $$E_{\mathrm{cell}}=\;E_{\mathrm{rev}}^{0}+\;\eta_{\mathrm{kin}}+\;\eta_{\Omega }+\;\eta_{\mathrm{mt}}$$

where *η*_kin_ is the kinetic, *η*_Ω_ is the Ohmic, and *η*_mt_ is the mass transport overpotential. Since hydrogen evolution reaction is more favorable in kinetics and mass transport under current testing conditions compared to oxygen evolution reaction (OER), the overpotential analysis only considered the OER side.

At a temperature of 80 °C, the saturation pressure of H_2_O was 0.47 bar_a_. For liquid water, the activity of water, *a*(H_2_O), was 1, while the activity of the gaseous species was represented by the ratio of their partial pressure to the standard pressure of 1 bar. The temperature‐dependent standard reversible potential, $E_{\mathrm{rev}}^{0}$, could be obtained from the literature^[^ ^36^ ^]^

(3) $$E_{\mathrm{rev}}^{0}=1.2291\;-0.0008456\cdot \left(T-298.15\right)$$

where the voltages, first two terms on right‐hand side of equation, were measured in V, and the temperatures in K. Under current electrolyzer testing condition, the $E_{\mathrm{rev}}^{0}$ was calculated to be 1.168 V, with a thermoneutral voltage of 1.42 V.

### Ohmic Overpotential *η*_Ω_

EIS was used to measure the high‐frequency resistance (HFR) representing the total electronic cell resistance *R*_tot_. The ohmic overpotential, *η*_Ω_, was therefore determined as

(4) $$\eta_{\Omega }=i*R_{\mathrm{tot}}=i*\mathrm{HFR}$$

### Kinetics Overpotential *η*_kin_

The kinetic overpotential was extracted using a Tafel model, in which the Tafel slope *b* and exchange current density *i*_0_ are the governing kinetic parameters. Assuming a nonpolarizable HER, the entire kinetic overpotential of the cell was governed by OER with the Tafel slope *b* as 2.303*RT/4F where *R* is the ideal gas constant, *T* is the temperature, and *F* is the Faraday's constant

(5) $$\eta_{\mathrm{kin}}=b*\log\log\;\left(\frac{i}{i_{0}}\right)$$

### Mass Transport Overpotential *η*_mt_

The mass transport is a summary of gaseous/liquid transfer in the PTL/CL and ionic transport in the CLs. In this study, it was calculated by subtracting the reversible cell potential and kinetic and Ohmic overpotentials from the measured cell potential.

### PTL Characterization

The PTLs were first subjected for mechanical testing by Nel/Hydrogen to examine the feasibility for commercial PEMWEs. A spanwise strength test was conducted to assess the ability of the PTL to withstand the forces applied under differential pressure conditions; all the PTLs showed adequate mechanical strength for electrolysis applications (Figure S1, Supporting Information).

### SEM and X‐Ray Micro Computed Tomography

Porous transport layer (PTL) surface morphology was imaged by an FEI Quanta FEG 250 SEM, Figure S2 in the Supporting Information. Ex situ XCT was conducted at the Advanced Light Source at Lawrence Berkeley National Laboratory (Beamline 8.3.2) with a PCO.Edge CCD camera, an LuAG scintillator, and 20x optical lenses. The resulting images had a resolution of 0.323 µm voxel^−1^ and a horizontal field of view of 1.8 mm. The samples were prepared for tomography imaging by cutting the PTLs into ≈3 mm triangular sections each having an ≈45° tip and 5 mm base for mounting on the pins which were mounted on the beamline rotating stage. A multilayer monochromator was used to select the X‐ray energy at 20 keV. 300 ms exposure time was used. Operando XCT and radiography imaging were conducted at Beamline 2‐BM at Advanced Photon Source (APS). A multilayer monochromator was used to select 27 keV energy. The optics used were Optique 2X lens, 20 um LuAGb scintillator, resulting in 1.73 um voxel^−1^ resolution. 20 ms exposure time was used for XCT data acquisition. Radiography imaging was used to capture oxygen transport in the channels. The data were collected with the cell in‐plane and through‐plane configuration, using 5 ms exposure time for a total time of 2 min per each operating condition.

### Modeling

A computational fluid dynamic (CFD) with the LBM was chosen to perform the numerical simulation of oxygen transport inside the PTL for PEMWE. 3D time‐dependent simulation with a multiphase flow model was used to predict the mass transport in various types of PTLs. This model used a lattice particles‐based instead of the traditional meshing method to generate the computational domain inside the complex structure of PTLs, which could reduce the processing time of mesh creation significantly. The Boltzmann transport equation allowed to develop a macroscopic model for modeling the transport phenomena in the computational domain. LBM was an appropriate CFD technique for solving fluid dynamics problems in the complex detailed structure of porous mediums such as PTLs. The PTL samples used in this simulation are shown in Figure 2. The computational domain had a size of 1000 × 1000 x *h* µm^3^, where *h* is the high of each sample. The lattice size or particle mesh size was set at 0.7 µm. The time step was set to 0.02 µs per time step. The land geometry was introduced into the middle of the computational domain with a width of 500 µm. The anode side of this PEMWE was assumed to be the control in this simulation. Through this modeling, the characteristic and transport behavior of the reactant and product were investigated under the operating condition of 0.5, 1.0, 2.0, and 3.0 A cm^−2^ with a flow rate of 2 sccm. The PTL surface wettability was assumed to be constant at 10°. The isothermal model was applied in this simulation. The boundary condition of each surface was defined and shown in Figure S10 in the Supporting Information. The commercial software XFlow 2020 Refresh 1 Beta (Build 108.07) was used to perform the calculations.

### Statistical Analysis

In this work, the polarization curves of PEMWEs for each porous transport layer were collected for at least three times and the voltage mean values at each current density were chosen as the final data to present. The software used in this work for simulation and data analysis was XFlow 2020 Refresh 1 Beta (Build 108.07). The software used for analyzing X‐ray CT data was ImageJ.

## Conflict of Interest

The authors declare no conflict of interest.

## Author Contributions

X.P. and N.D. conceived the idea and designed the experiments. N.D. and I.Z. obtained funding to support the work and provided experimental design guidance and direction. X.P. and Z.T. carried out the electrochemical measurements and analysis. P.S. and I.Z. performed the synchrotron X‐ray tomography experiments and modeling. C.C., A.K., and L.W. performed the PTL characterization. X.P., N.D., I.Z., and P.S. wrote the manuscript, and all authors edited the manuscript.

## Supporting information

Supporting Information

## Data Availability

The data that support the findings of this study are available from the corresponding author upon reasonable request.
