# Supplementary material for: Insights into Interfacial and Bulk Transport Phenomena Affecting Proton Exchange Membrane Water Electrolyzer Performance at Ultra‐Low Iridium Loadings
Source: Adv Sci (Weinh). 2021 Sep 26;8(21):2102950. doi: 10.1002/advs.202102950 (PMC8564452; doi:10.1002/advs.202102950)
Supplement: Supplementary file 1 — Supporting Information [file ADVS-8-2102950-s001.pdf]

© 2021 Wiley-VCH GmbH

ADVANCED SCIENCE
Open Access

Supporting Information

for *Adv. Sci.*, DOI: 10.1002/advs.202102950

Insights Into Interfacial and Bulk Transport Phenomena Affecting Proton Exchange Membrane Water Electrolyzer Performance at Ultra-low Iridium Loadings

Xiong Peng[1], Pongsarun Satjaritanun[2], Zachary Taie[1], Luke Wiles[3], Alex Keane[3], Christopher Capuano[3], Iryna V. Zenyuk[2], and Nemanja Danilovic[1*]*

Insights Into Interfacial and Bulk Transport Phenomena Affecting Proton Exchange Membrane Water Electrolyzer Performance at Ultra-low Iridium Loadings

Xiong Peng[1], Pongsarun Satjaritanun[2], Zachary Taie[1], Luke Wiles[3], Alex Keane[3], Christopher Capuano[3], Iryna V. Zenyuk[2*], and Nemanja Danilovic[1*]

[1] Energy Storage and Distributed Resources Division, Lawrence Berkeley National Laboratory, Berkeley,

CA, 94720, USA

[2] Department of Material Science and Engineering, University of California Irvine, Irvine, CA, 92697, USA

[3] Nel Hydrogen/Proton Onsite, Wallingford, CT, 06492, USA

Corresponding author:

iryna.zenyuk@uci.edu

ndanilovic@lbl.gov

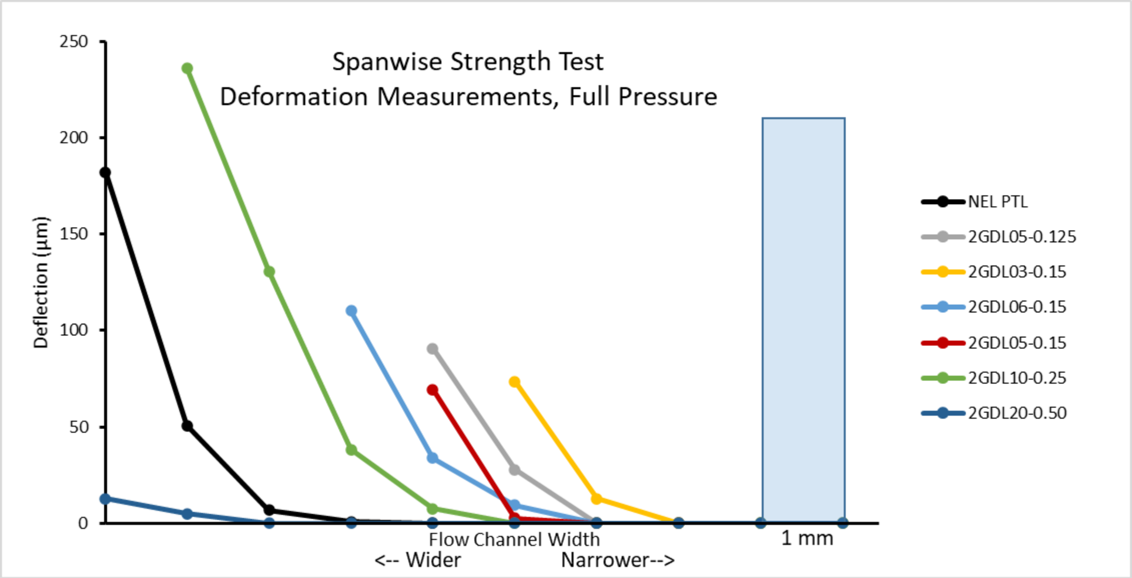

Figure S1. Spanwise strength test showing the deflection of PTLs as a function of channel width. Blue bar marks target span width for FCT parallel channel flow field width. Full pressure refers to 30 bar H_2 pressure plus active area pressure and safety factors which are proprietary cell design information.

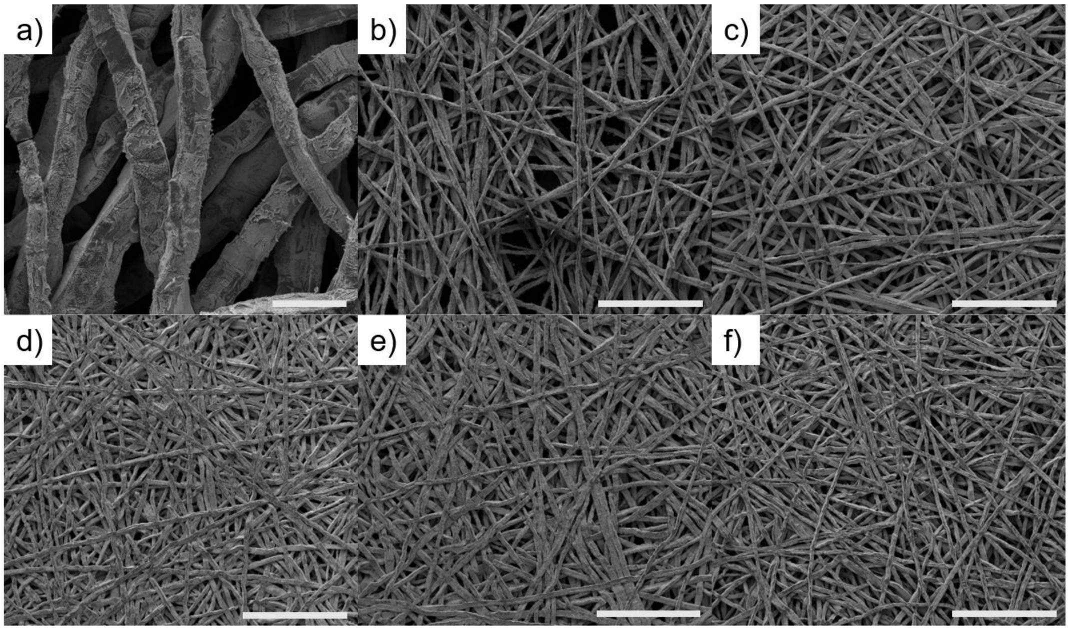

Figure S2. Scanning electron microscopy of the surface morphology of Ti fiber based PTL a) fiber diameter, scale bar: 50 µm, b)-f) 03-0.15, 05-0.15, 06-0.15, 05-0.125 and 10-0.25, scale bar: 500 µm.

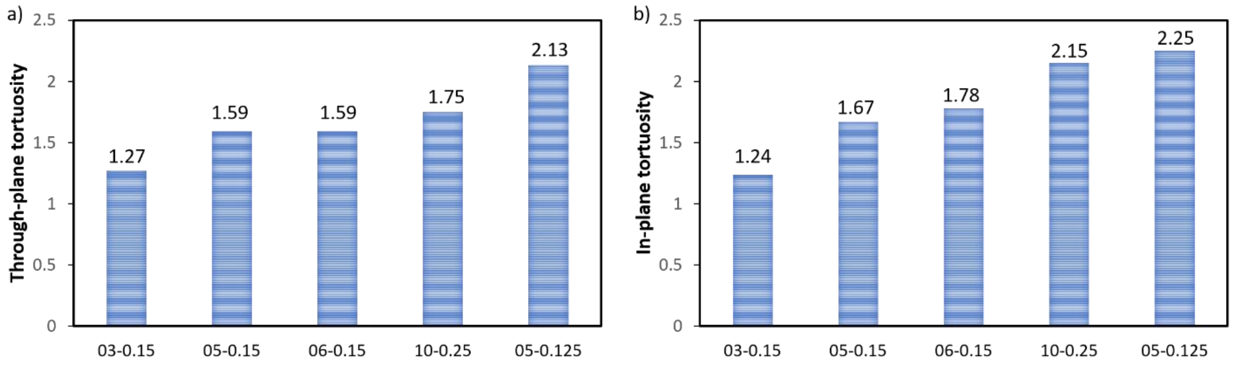

Figure S3. Through-plane and in-plane tortuosity of the PTLs. Tortuosity calculated from X-ray CT measurements.

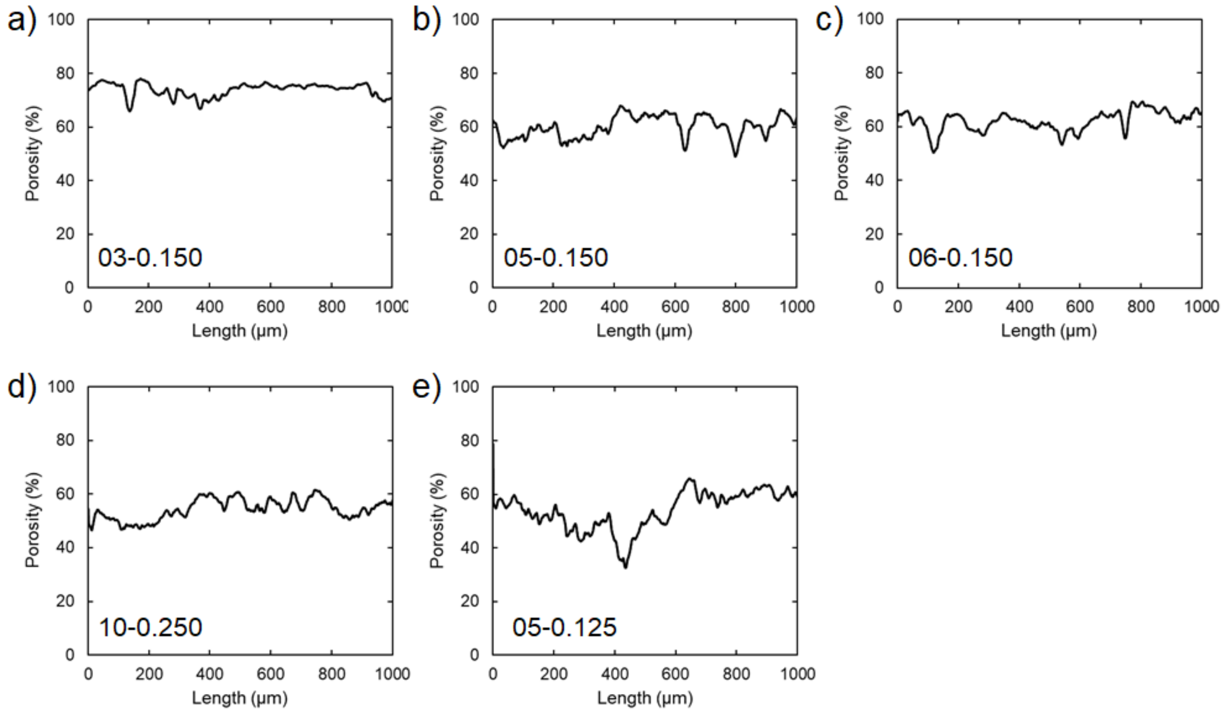

Figure S4. In-plane porosity profiles of PTL sample a) 03-0.150, b) 05-0.150, c) 06-0.150, d) 10-0.250, and e) 05-0.125.

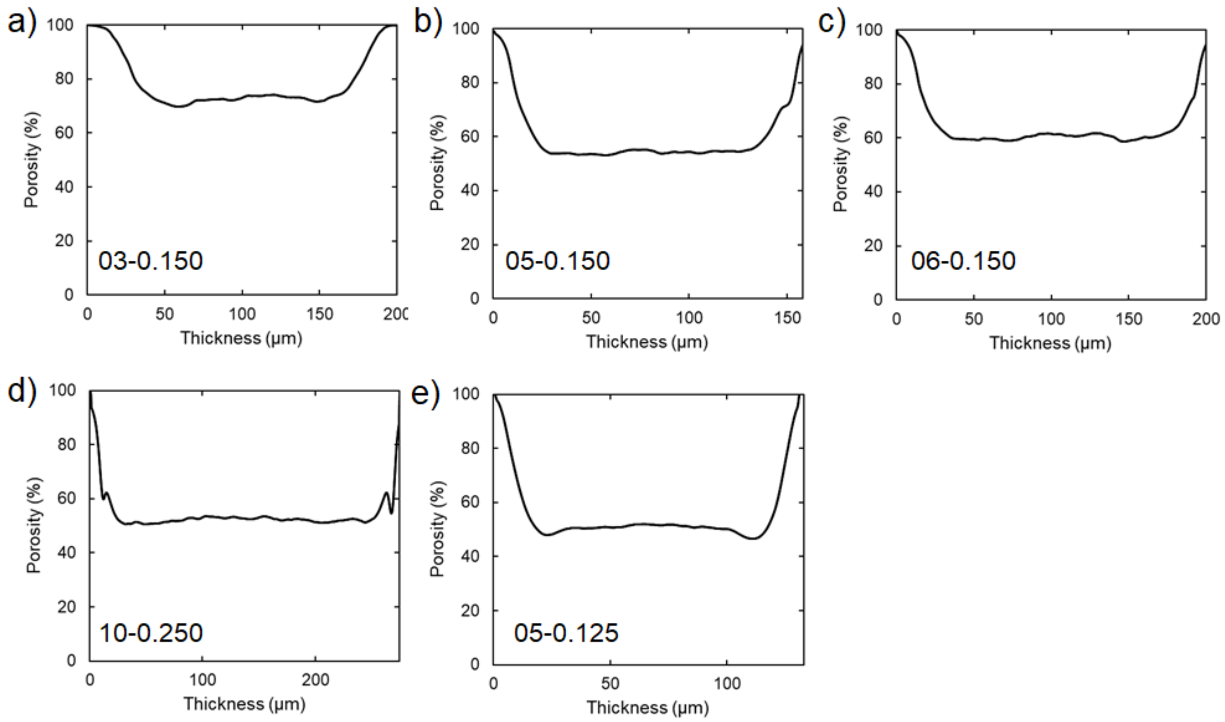

Figure S5. Through-plane porosity profiles of PTL sample a) 03-0.150, b) 05-0.150, c) 06-0.150, d) 10-0.250, and e) 05-0.125.

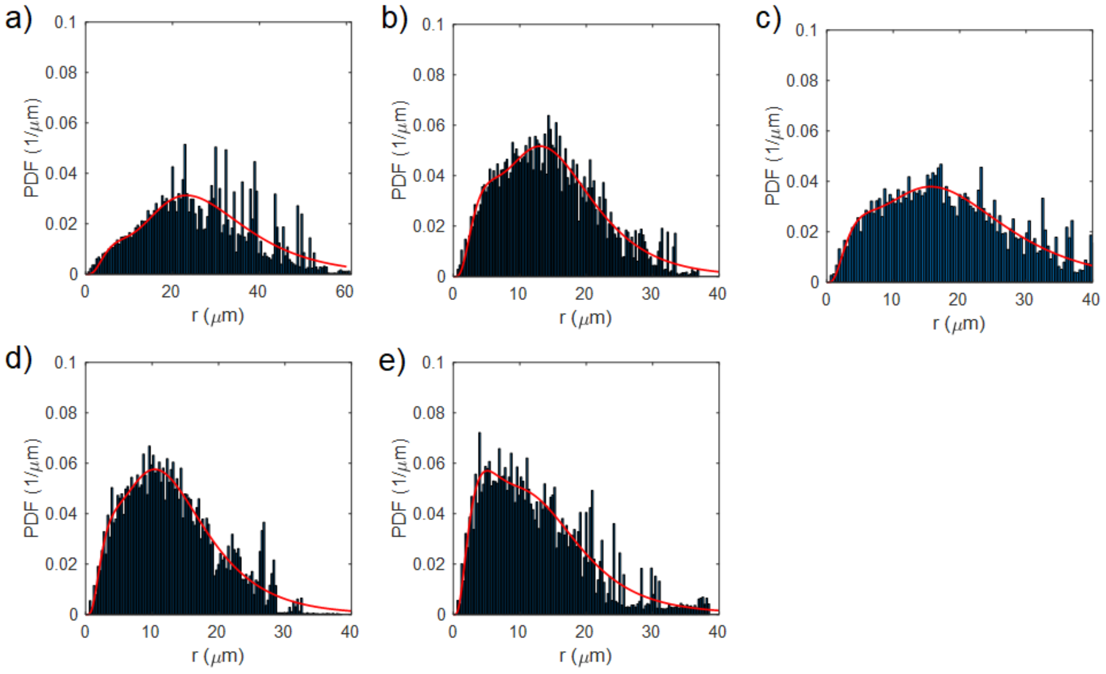

Figure S6. Pore-size distribution of PTL sample a) 03-0.150, b) 05-0.150, c) 06-0.150, d) 10-0.250, and e) 05-0.125.

Contact area calculations were performed on an interfacial area of 1 mm^2. 5 slices were selected near the interface corresponding to about 8.6 µm in length to accommodate the membrane swelling into the PTL during operation. The catalyst was thresholded within these slices and a Z-projection was done to superimpose it into one single slice. The area fraction of the catalyst slice was calculated and divided by the area fraction of the PTL slice at the interface to obtain the triple phase contact area (TPCA%), Figure S7. Three such representative areas were selected randomly and the TPCA% was calculated at each one to get error bars.

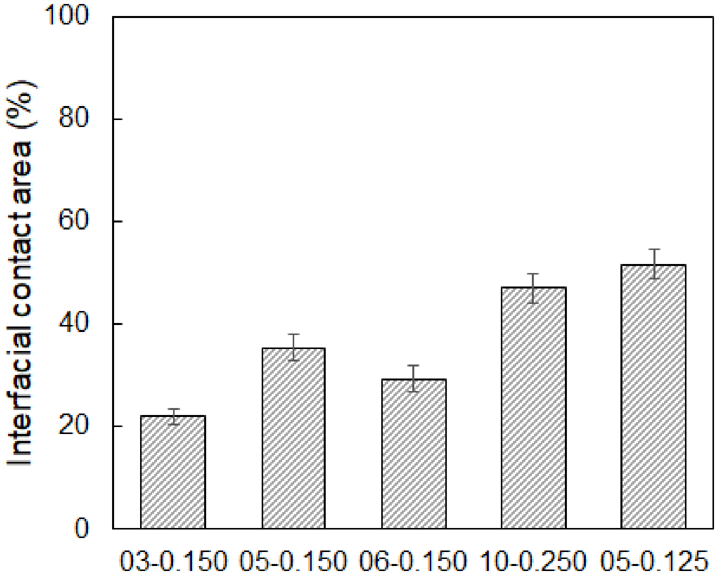

Figure S7. Interfacial contact area of the PTL/CL interface for all PTL samples.

By comparison, PTL 03-0.15 has the highest porosity and lowest tortuosity, PTL 05-0.15 and 06-0.15 have intermediate porosity and tortuosity while PTL 10-0.250 and 05-0.125 have the lowest porosity and highest tortuosity (Figures S3-S6). In-plane and through-plane porosity distributions are shown in Figure S5 and S6, where relatively uniform porosity distributions were observed through-plane, however the PTLs with low porosity showed inhomogeneous through-plane porosity distributions. In-plane tortuosity values are found to be higher than the through-plane tortuosity for all the PTLs except for 0.3-0.150 (Figure S3). In contrast the through-plane tortuosity is more consistent, indicating that in-plane there might be some dead-ended pores that block transport. The PTL mean pore size follows the trend for porosity, PTLs with higher porosity also had larger average pore sizes (Figure S6). For example, for 03-0.150 with a porosity of 74 % the average pore radius is 28 µm, whereas for 10-0.250 PTL, which has the lowest porosity of 54.4 %, the average pore radius was reduced by more than half to 13.4 µm. The overall pore size distributions are shown in Figure S7, where either bimodal or unimodal distributions of pores are observed. The PTL thicknesses were around 150 µm for PTL's 03-0.150, 05-0.150 and 06-0.150, while thickness for PTL 10-0.250 and 05-0.125 is 250 µm and 122 µm, respectively.

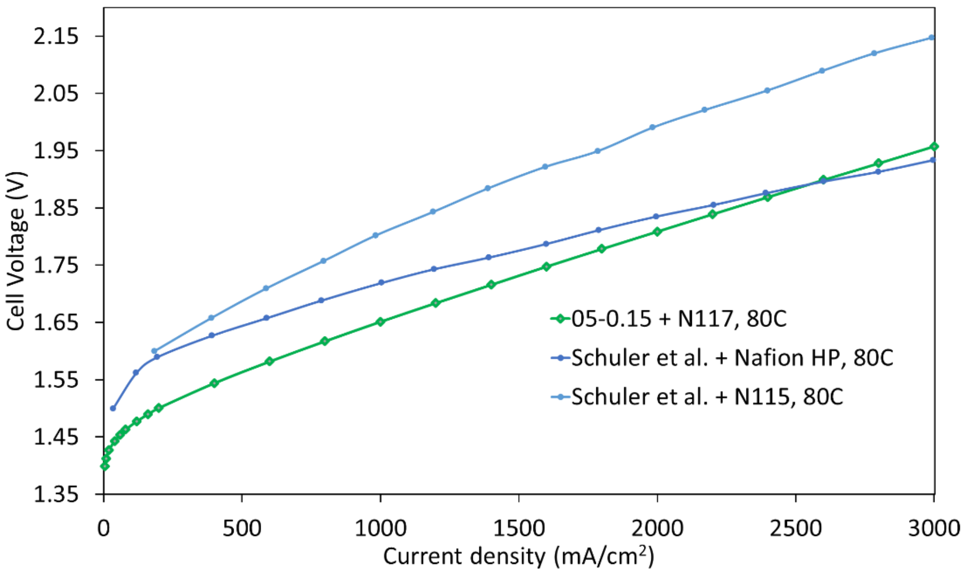

Figure S8. Comparison with literature data on microporous layer (MPL) containing PTLs.

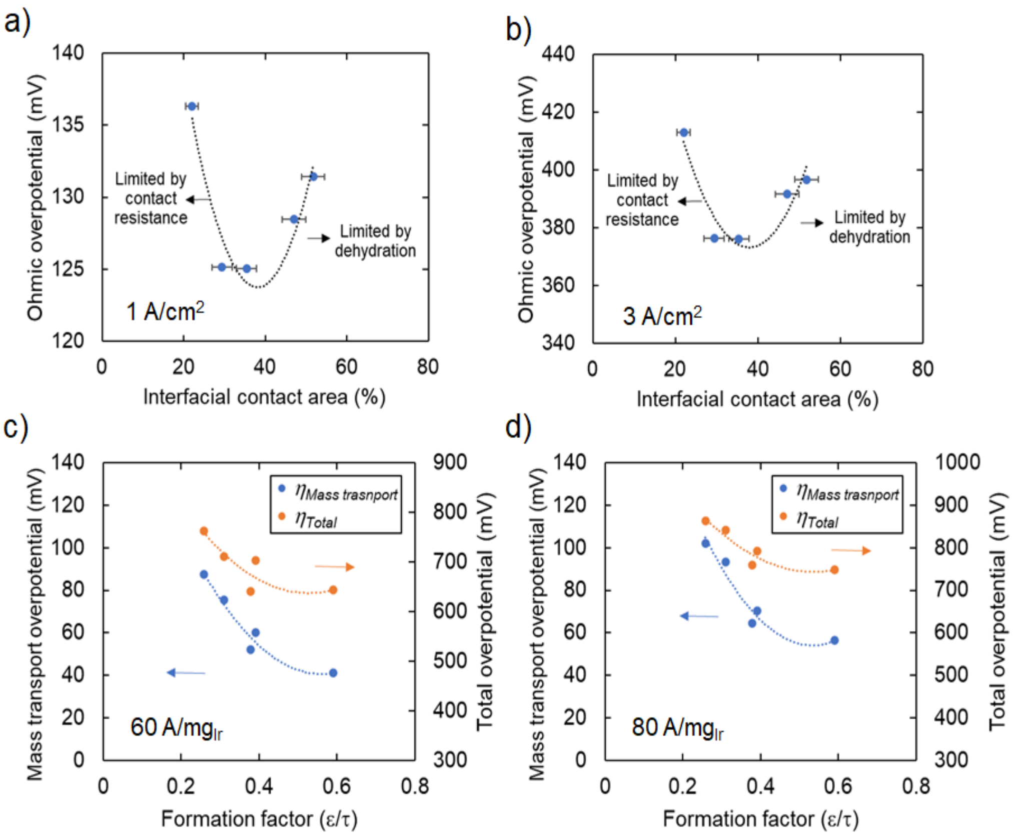

Figure S9. Effect of interfacial contact area on the ohmic overpotential at a) 1 A/cm^2, and b) 3 A/cm^2. effect of formation factor on mass transport overpotential and total overpotential at c) 60 A/mg$_{Ir}$, and d) 80 A/mg$_{Ir}$.

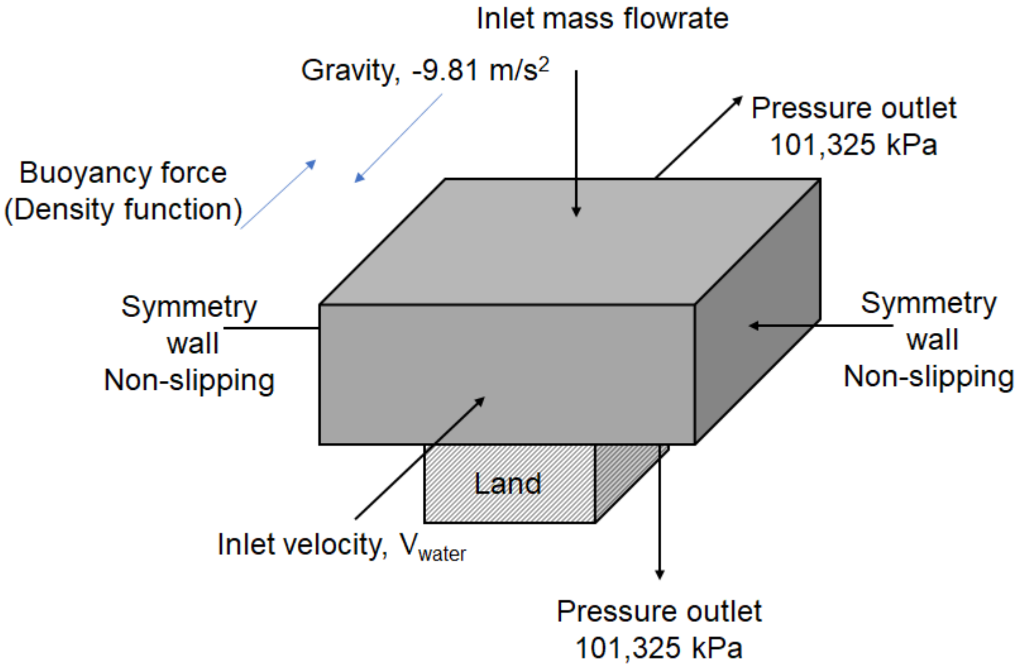

Figure S10. Schematic representation of LBM modeling boundary conditions setup.

Figure S11 shows the oxygen flux modeling through the PTL at pore areas (non PTL domain). The oxygen flux for a given current density is calculated from Faraday's law, as shown by Equation S6 in Figure S11. The flux is also represented in terms of the active site or oxygen nucleation site, which depends on the catalyst coating, loading, and its distribution. In this model, the oxygen gas was created as a bubble and nucleated within the nucleation sites of the catalyst layer.[2] The model assumed the nucleation site has a nucleation radius of 1 µm with the nucleation density of 1 site/µm. The shape and size of oxygen bubbles can be characterized by using the Eotvos number, $\square_0$ ($\square_0 = \frac{\square\square\square\square^2}{\square}$), which is correlated to the surface tension (σ), bubble characteristic diameter ($\square$), and density between the fluids($\Delta\rho$), and gravity ($\square$). Once the oxygen bubble nucleates, its radius will grow to a critical size and it will detach from the nucleation site, then it has the available spaces that allow liquid water to reach the active site at the catalyst layer. After that, the next bubble will nucleate and grow and the process will repeat. The pink area on the left of Figure S11 is the pore contact area (A_{eff}), which is essentially a non-PTL contact area. If there is a perfect distribution of catalyst, due to uniform coating and all catalyst sites are active, the following holds true: $\varnothing_{site}$ = 1 (i.e, or $\varnothing_{site}$ = 0.75 when 75% of catalyst sites are active). Here we assume $\varnothing_{site}$ = 1. The effective oxygen flux was calculated by using Equation S7. In this work, the modeling was done to operate the cell at 1, 2, and 3 A/cm^2, then we can calculate the oxygen flux based on the open pore area. The bar chart below shows the oxygen flux per 1 mm^2 area at 1 A/cm^2 at the water flow rate of 2 sccm.

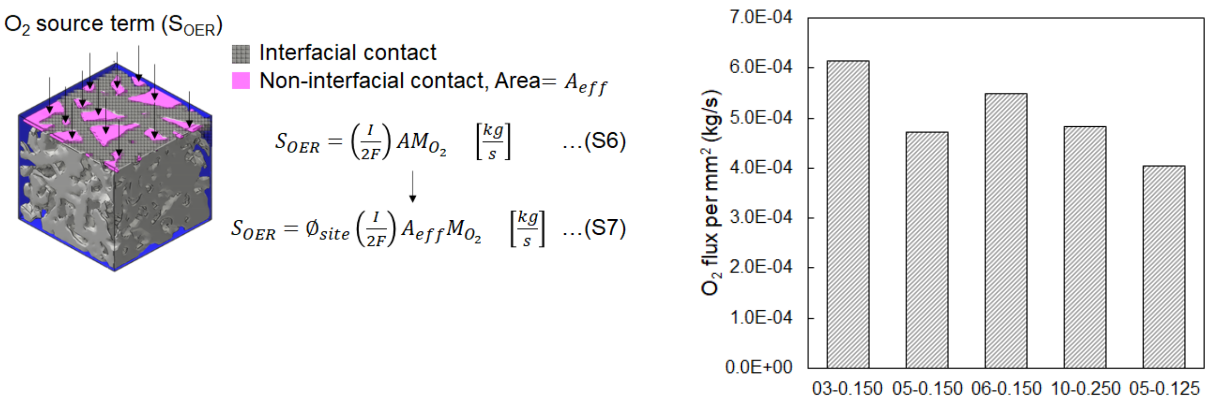

Figure S11. Oxygen flux modeling through PTL non-interfacial contact areas.

To obtain the oxygen content distribution profiles, the oxygen content is calculated from the area fraction of oxygen content ($\square_{O_2}$) and PTL pore space ($\square_{\square\square\square\square}$) in each slice of the PTL portions. Since oxygen only exists in a gas phase, one can calculate the amount of area that is occupied by oxygen vs. water. So if a pore is completely filled by oxygen Φ_{O_2}= 1, on the other hand, if the pore is filled by water, Φ_{O_2}= 0. In this work, we have separated the PTL into 3 portions, which represent the interface of CL/PL, middle of PTL, and PTL/channel. In each portion, the combined stack containing 10 top re-slices was used to calculate the oxygen content. The Z-project (ImageJ) was taken through-thickness oxygen content calculation, visualizing, and mapping the oxygen content distribution. The oxygen content (Φ_{O_2}) is calculated by the following equation:

$$\Phi_{O_2} = \frac{\square_{O_2}}{\square_{\square\square\square\square}} \qquad \textit{Equation S5}$$

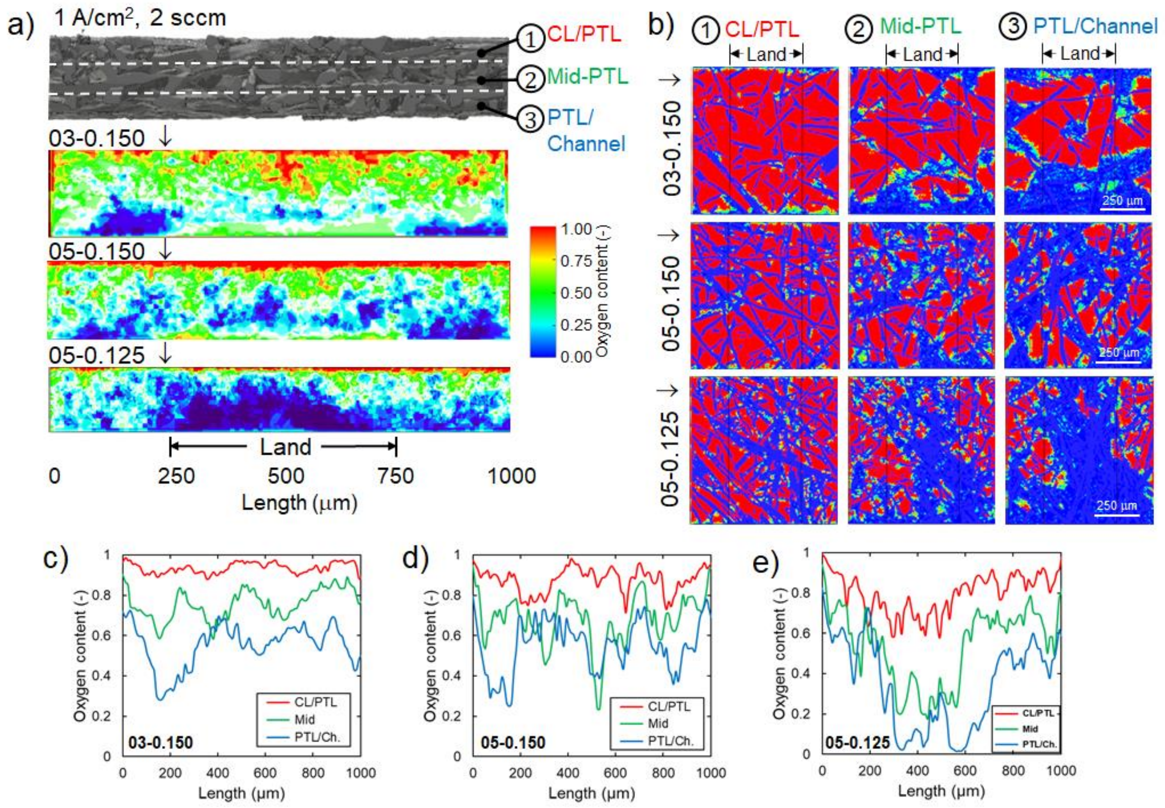

Figure S12. Summary plots detailing the oxygen distribution at interface of CL/PTL, middle interface of PTL, and interface of PTL/flow field channel for the samples of 03-0.15, 05-0.15, and 05-0.125, respectively. a) In-plane oxygen distribution of these samples, b) Through-plane oxygen distribution at each interface. LBM simulation was conducted at 1 A/cm^2, and oxygen content profiles comparison within different PTL portions as a function of distance for sample c) 03-0.15, d) 05-0.15, and e) 05-0.125.

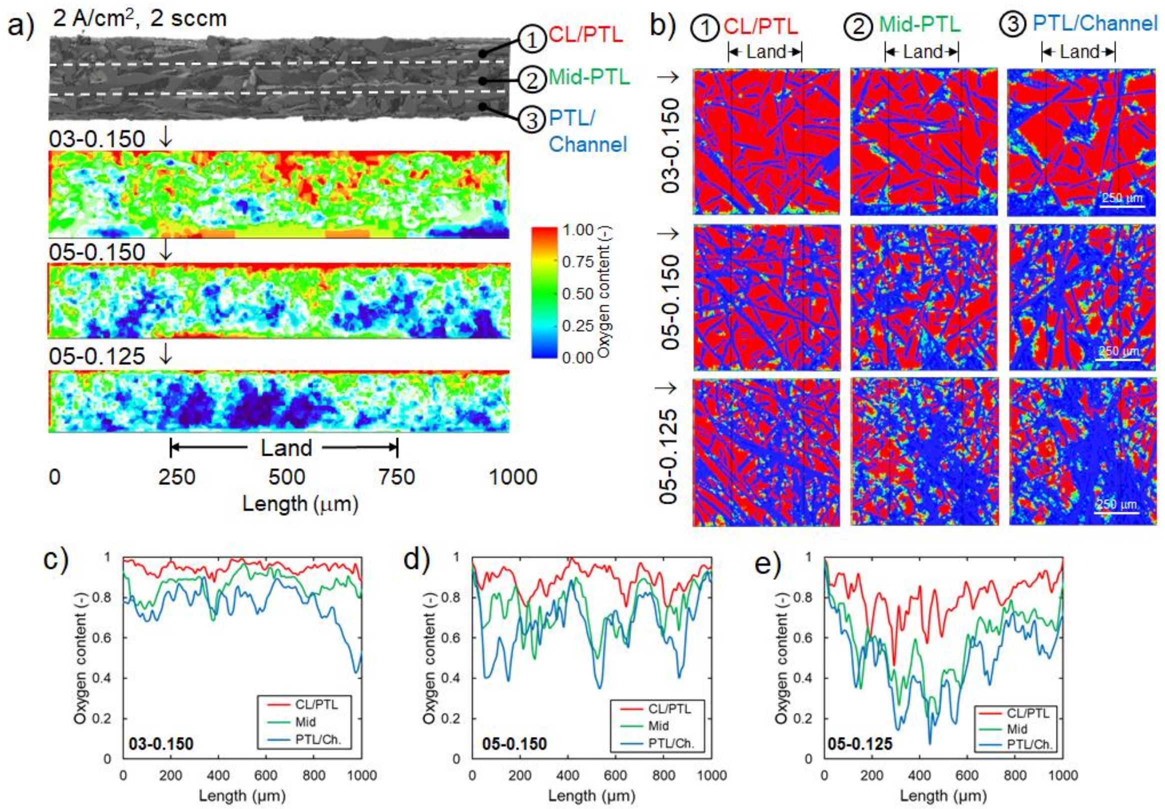

Figure S13. Summary plots detailing the oxygen distribution at interface of CL/PTL, middle interface of PTL, and interface of PTL/flow field channel for the samples of 03-0.15, 05-0.15, and 05-0.125, respectively. a) In-plane oxygen distribution of these samples, b) Through-plane oxygen distribution at each interface. LBM simulation was conducted at 2 A/cm^2, and oxygen content profiles comparison within different PTL portions as a function of distance for sample c) 03-0.15, d) 05-0.15, and e) 05-0.125.

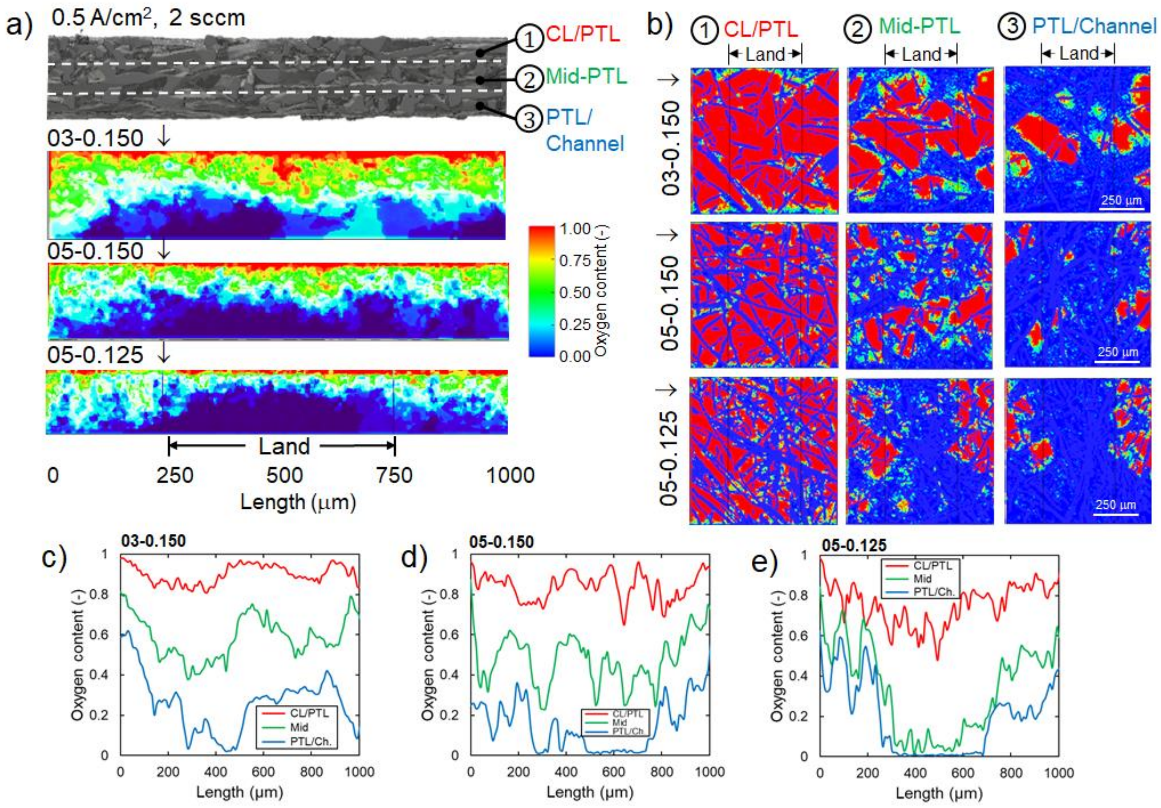

Figure S14. Summary plots detailing the oxygen distribution at interface of CL/PTL, middle interface of PTL, and interface of PTL/flow field channel for the samples of 03-0.15, 05-0.15, and 05-0.125, respectively. a) In-plane oxygen distribution of these samples, b) Through-plane oxygen distribution at each interface. LBM simulation was conducted at 0.5 A/cm^2, and oxygen content profiles comparison within different PTL portions as a function of distance for sample c) 03-0.15, d) 05-0.15, and e) 05-0.125.

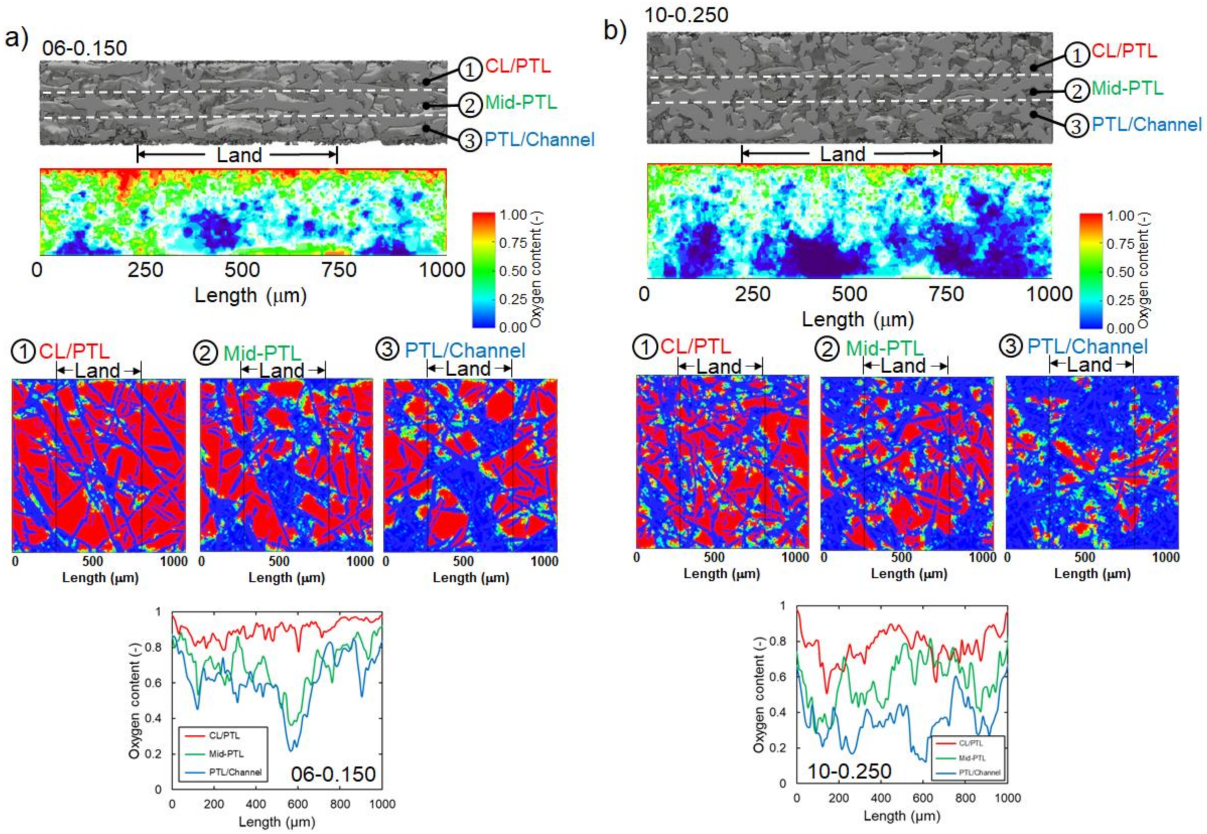

Figure S15. Lattice Boltzman modeling results at 1 A/cm2 of a) 06-0.15 and b) 10-0.25 PTLs.

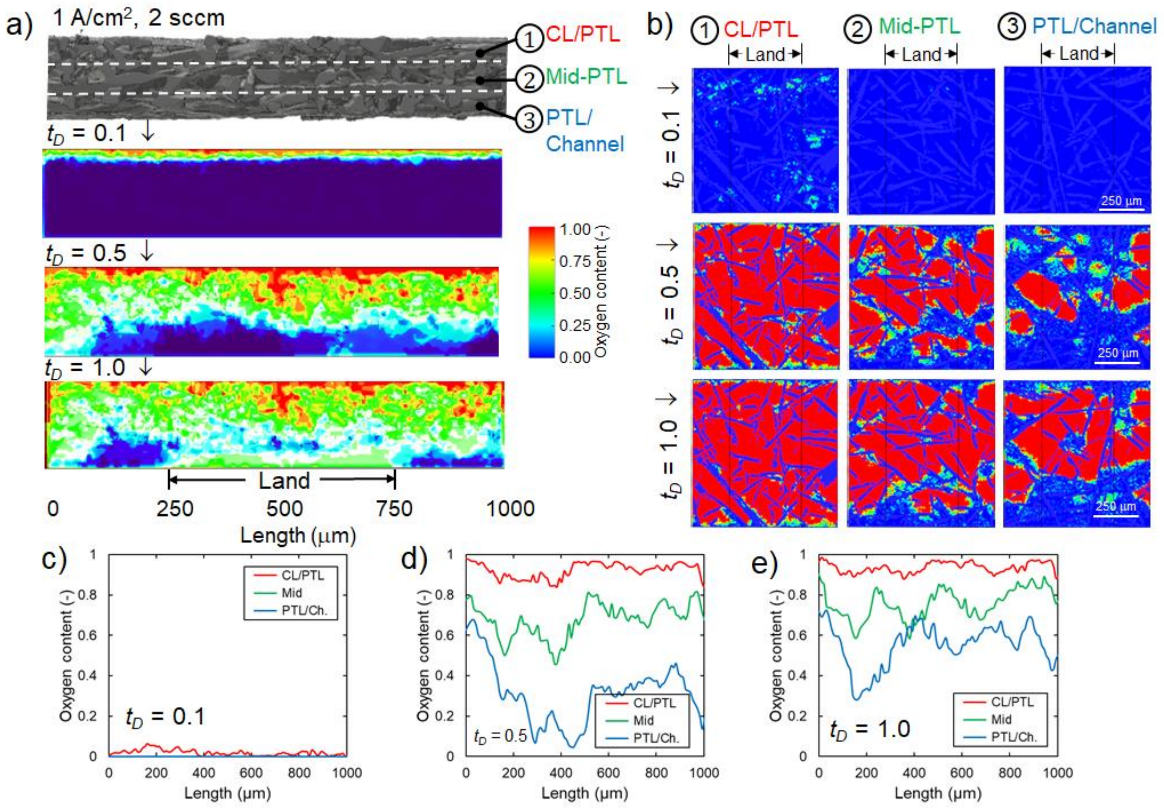

Figure S16. Oxygen content distribution averaging over the time of sample 03-15 at the current density of 1 A/cm2 with the water flow-rate of 2 sccm. Note that, t_D is dimensionless time.

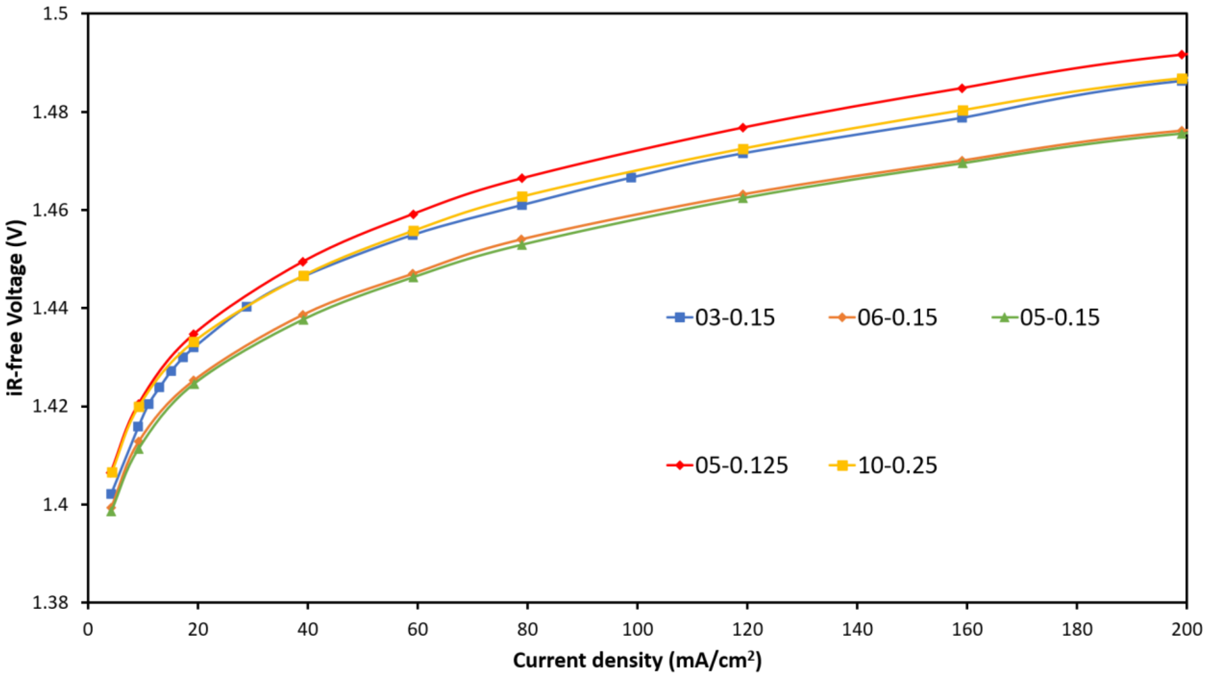

Figure S17. The comparison of iR-free voltage of different PTLs at low current densities.

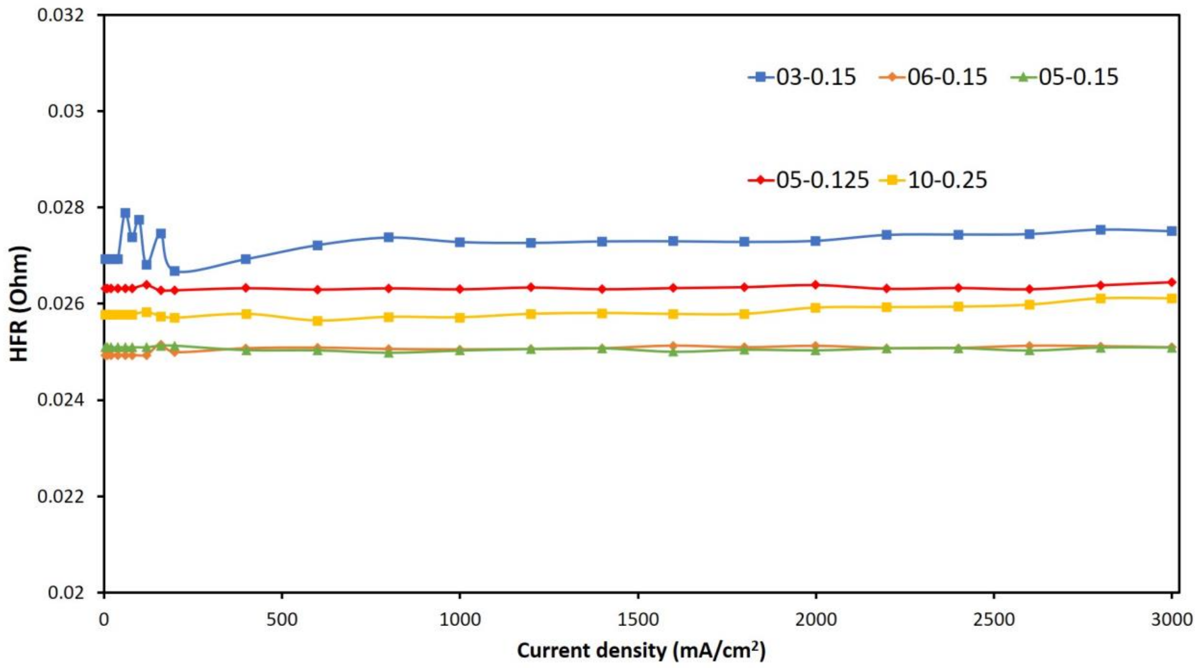

Figure S18. The high frequency resistance (HFR) recorded during PEMWE operation for different PTLs. Electrode geometric surface area is 5 cm^2.

Table S1. The comparison of PEMWE performance between this work and what reported in the literature

Reference	Ir loading (mg_{Ir}/cm^2)	Membrane Type	Operating Temperature (℃)	Cell Voltage at $2A/cm^2$ (V)	Cell Voltage at $3A/cm^2$ (V)
**This work**	0.030	N117	80	1.81	1.95
1	0.4	E100-09S	80	1.75	1.88
2	0.1	N212	90	1.85	>2
3	0.1	N212	90	1.88	>2
4	3.0	N212	80	1.7	---
5	0.02	N212	80	1.9	---
6	0.2	N212	80	1.82	1.95
7	0.12	N115	80	1.9	---
8	2.5	N115	60	1.95	2.15
9	2	N115	80	1.9	2.0
10	0.1	N117	80	1.85	>2

Table S2. The High Frequency Resistance (HFR) used at current range of 4 - 60 **mA/cm²** for Tafel Plot fitting and the obtained Tafel Slope and Apparent Exchange Current density for different PTLs.

PTL type	Tafel Slope (mV/dec)	HFR Used (Ohm)	Apparent Exchange Current Density (mA/cm²)
**03-0.15**	46.4	0.026927	$3.93*10^{-5}$
**05-0.15**	41.6	0.025093	$1.27*10^{-5}$
**06-0.15**	41.2	0.024933	$1.05*10^{-5}$
**10-0.25**	43.9	0.02577	$1.65*10^{-5}$
**05-0.125**	45.5	0.02632	$1.68*10^{-5}$

Reference:

1. Siracusano, S., Baglio, V., Van Dijk, N., Merlo, L. & Aricò, A. S. Enhanced performance and durability of low catalyst loading PEM water electrolyser based on a short-side chain perfluorosulfonic ionomer. *Appl. Energy* **192**, 477–489 (2017).

2. Lee, B. S. *et al.* Development of electrodeposited IrO2 electrodes as anodes in polymer electrolyte membrane water electrolysis. *Appl. Catal. B Environ.* **179**, 285–291 (2015).

3. Lee, B. S. *et al.* Polarization characteristics of a low catalyst loading PEM water electrolyzer operating at elevated temperature. *J. Power Sources* **309**, 127–134 (2016).

4. Su, H., Bladergroen, B. J., Linkov, V., Pasupathi, S. & Ji, S. Study of catalyst sprayed membrane under irradiation method to prepare high performance membrane electrode assemblies for solid polymer electrolyte water electrolysis. *Int. J. Hydrogen Energy* **36**, 15081–15088 (2011).

5. Park, J. E. *et al.* Ultra-low loading of IrO 2 with an inverse-opal structure in a polymer-exchange membrane water electrolysis. *Nano Energy* **58**, 158–166 (2019).

6. Hegge, F. *et al.* Efficient and Stable Low Iridium Loaded Anodes for PEM Water Electrolysis Made Possible by Nanofiber Interlayers. *ACS Appl. Energy Mater.* **3**, 8276–8284 (2020).

7. Rozain, C., Mayousse, E., Guillet, N. & Millet, P. Influence of iridium oxide loadings on the performance of PEM water electrolysis cells: Part II - Advanced oxygen electrodes. *Appl. Catal. B Environ.* **182**, 123–131 (2016).

8. Suermann, M. *et al.* Femtosecond laser-induced surface structuring of the porous transport layers in proton exchange membrane water electrolysis. *J. Mater. Chem. A* **8**, 4898–4910 (2020).

9. Schuler, T. *et al.* Hierarchically Structured Porous Transport Layers for Polymer Electrolyte Water Electrolysis. *Adv. Energy Mater.* **10**, 1903216 (2020).

10. Alia, S. M., Stariha, S. & Borup, R. L. Electrolyzer Durability at Low Catalyst Loading and with Dynamic Operation. *J. Electrochem. Soc.* **166**, F1164–F1172 (2019).
